# Protease cleavage at an engineered tetra-basic motif in Drosophila PTTH accelerates developmental timing

**DOI:** 10.17912/micropub.biology.000168

**Published:** 2019-09-26

**Authors:** MaryJane Shimell, Michael B O'Connor

**Affiliations:** 1 Department of Genetics, Cell Biology and Development, University of Minnesota, Minneapolis MN 55455

**Figure 1:**
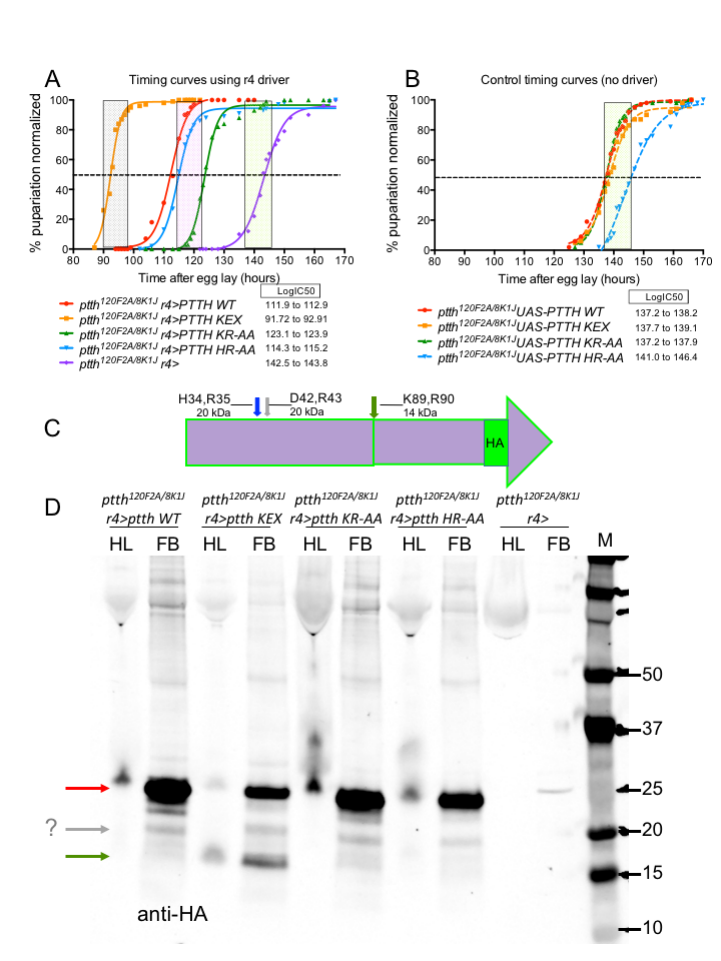
Loss of HR or KR sites does not affect PTTH activity but conversion of KR to a tetrabasic cleavage site enhances PTTH activity: (A) Developmental timing curves of different PTTH species: WT (red), KEX (orange), KR-AA (green), HR-AA (blue) and control fat body driver (purple). The dashed line indicates 50% pupariation and the calculated value is the LogIC50. The vertical shaded bars denote an 8-hour window for delayed (green shading), wild type (pink shading) and accelerated (grey shading) timing. (B) Developmental timing curves for the driverless UAS controls illustrates the 1-day delay observed in *ptth* mutants. (C) Cartoon of the PTTH cDNA used in these studies. The HR (blue arrow) and KR (green arrow) residues that were mutated are shown and the predicted size of the processed PTTH protein is noted. A third candidate site for processing, DR (grey arrow), which was not mutated in this study, is noted along with its predicted size. (D) Western blot of hemolymph (HL) and fat body (FB) from wandering third instar larvae probed with HA antibody. Due to high protein content of HL, samples migrate with some bulleting. Full length PTTH-HA migrates at 24 kDa (red arrow) and is found in both HL and FB. Note that neither KR-AA nor HR-AA mutations lose their predicted proteolytic fragments from the WT sample (14 kDa and 20 kDa, respectively). Also note that when KR is mutated to RRKR, a 14 kDa protein is observed (green arrow) in both HL and FB. Lastly a 20 kDa protein, very weak in intensity, is seen in all samples (grey arrow) and may represent DR processed PTTH.

## Description

In *Drosophila melanogaster*, the neuropeptide prothoracicotropic hormone (PTTH) is a modulator of metamorphic timing and aids in adaptation to environmental challenges (Shimell et al. 2018). The *ptth* gene can be found in most insect species, with the exception of Apidae (Skelly et al. 2019), suggesting conservation of structure and function. PTTH binds to its receptor Torso, a receptor tyrosine kinase, to activate a MAPK signaling pathway, which ultimately regulates transcription of the ecdysone biosynthetic genes and the production of ecdysone (McBrayer et al. 2007, Rewitz et al. 2009). Trunk, a paralog of PTTH, also binds Torso, but unlike PTTH, Trunk signals in the early embryo to specify termini cell fates (Duncan et al. 2013). It has been shown that the pro-domain of Trunk is cleaved intracellularly by Furin 1 and Furin 2 at amino acids K75,R76 and that processing is essential for terminal patterning (Henstridge et al. 2014). While prior *in vivo* and *in vitro* experiments in *Antheraea pernyi* (Sauman and Reppert 1996), *Manduca sexta* (Gilbert et al. 2000), and *Helicoverpa armigera* (Wei et al. 2005) demonstrated that the recombinant mature fragment of PTTH has the same activity as brain extracts, in each of these cases the negative control was not the pre-propeptide. Only in *Bombyx mori* has it been shown that mature PTTH, and not the pre-propeptide, is the active species (O’Reilly et al. 1995). Based on these observations, it has been generally accepted that pro-domain processing of PTTH is essential for function. For this reason, we wished to determine if, like Trunk, Drosophila PTTH also requires proteolytic cleavage of the pro-domain to be active.

Two dibasic residues in PTTH, H34,R35 (HR) and K89,R90 (KR), were predicted to be candidates for proteolytic processing (similarity to Trunk site and ProP 1.0 Server, Duckert et al. 2004). Wild type PTTH cDNA, as well as mutated versions of the putative processing sites, were 3XHA tagged at the C-terminus, cloned into *pUAST attB*, and integrated by phiC31 at the *attP2* site on the third chromosome. The putative processing sites were mutated to two alanines (HR-AA and KR-AA); additionally, the KR site was mutated to tetra-basic RRKR (“KEX”) to better match an optimal Furin cleavage site. The *ptth^8K1J^* mutation was built into each of the *UAS-PTTH* lines, and the *ptth^120F2A^* mutation was also introduced to the fat body driver line *r4-Gal4*.

The assay for PTTH activity involved looking for rescue of the one-day developmental timing delay of *ptth* mutants. We find that when either of the candidate processing sites are mutated, rescue of the timing defect still occurs, suggesting that proteolytic processing at these sites is not required for PTTH activity. Interestingly, when the KR site is converted to a more optimal Furin recognition sequence RRKR (KEX), timing is not only rescued, but accelerated approximately one additional day relative to wild type.

In order to identify the PTTH species found in hemolymph and fat body, samples were obtained from wandering third instar larvae and analyzed on Western blots. We observe the predicted full length 24 kDa protein in wild type PTTH and the three mutants, predominantly in the fat body, as well as a minor 20 kDa fragment in all lines and a new strong 14 kDa fragment in the KEX mutated PTTH line. These results suggest that efficient cleavage is not occurring at the two computer identified sites, which are likely not biologically relevant. However, if one of those sites, KR, is mutated to make a more optimal Furin cleavage site resulting in a new processed protein species, then the biological activity of PTTH is significantly enhanced as evidenced by the acceleration in time to pupation. Using a new prediction algorithm (PROSPERous, Song et al. 2018), we find another possible Furin cleavage site at D42,R43 which could produce the minor 20 kDa fragment seen in all lanes. Future work will entail mutating D42,R43 in order to test if Furin cleavage at this site is required for PTTH rescue. Taken together these results show that proteolytic processing of PTTH at either of two tested dibasic sites is not essential for activity. Perhaps cleavage at D42,R43 is required for wild type activity, since we find that engineering a new tetra-basic cleavage site results in higher PTTH activity, which is consistent with the idea that proteolytic processing is required.

## Reagents

w1118 ; ptth120F2A/CyO, P{w[+C]=ActGFP} ; r4-Gal4/TM3, P{w[+mC]=ActGFP}Ser[1]

w1118 ; ptth8K1J ; {UAS-PTTH 3XHA WT} attP2

w1118 ; ptth8K1J ; {UAS-PTTH 3XHA KEX} attP2

w1118 ; ptth8K1J ; {UAS-PTTH 3XHA KR-AA} attP2

w1118 ; ptth8K1J ; {UAS-PTTH 3XHA HR-AA} attP2

*yw ; ptth120F2A* (Shimell et al. 2018)

*yw ; ptth8K1J* (Shimell et al. 2018)

*P{w[+mC]=r4-GAL4}3* BDSC 33832

anti-HA C29F4 (rabbit mAb, Cell Signaling 3724, 1:2500 dilution)

goat anti-rabbit IRDye680 (VWR 102673-410, 1:10,000 dilution)

## Methods

Developmental timing curves. Culture conditions and timing curve derivations are described in Shimell et al. with these modifications. Newly ecdysed, GFP negative L1 larvae were transferred to food vials at a density of ~35 larvae/vial. Pupariation data from 4 individual vials was pooled and analyzed in GraphPad Prism using non-linear regression curve fit. Developmental time is that time at which 50% pupariation occurs or logIC50 of the regression curve.

Hemolymph and fat body collection. Hemolymph (HL) from wandering L3 larvae, cultured identically to the timing curves, was harvested by tearing open 5-6 larvae, aspirating the HL with a P2 pipette and placing in 25 μL ice cold PBS. Between 2 and 4 μL of HL were pooled in each of 4 biological replicates. Samples were spun at 10K 10 minutes at 4°. The supernatant was removed using a gel loading tip, sample buffer was added to a 2X concentration (125mM Tris pH 6.8, 20% (v/v) glycerol, 4% (w/v) SDS, 100 μg/mL bromophenol blue, 10% (v/v) β-mercapto-ethanol), heated at 95° 5 minutes and then stored at -40 until use. Additional 2X sample buffer was added to HL samples prior to loading gel to ameliorate bulleting of the sample.

Fat body (FB) was teased away from the carcasses of dissected wandering L3 larvae, making sure to avoid salivary glands, Malpighian tubules, and guts. Four biological replicates of the combined FBs of 3-4 larvae in 25 μL PBS were brought to 2X in sample buffer, heated at 95° 5 minutes and stored at -40 until use.

The four biological replicates of HL and FB were analyzed by Western blot, and the best sample was chosen for the composite Western.

Western blots. NuPAGE™ 4-12% Bis-Tris gels were run in MES SDS running buffer, blotted onto nitrocellulose, pre-hybridized and hybridized to antibodies under conditions recommended by Life Technologies and Li-Cor. Membrane was scanned using the Li-Cor Odyssey instrument with scan parameters of 84 μm resolution, 5.0 intensity and medium quality.
